# Efficacy of *Trichoderma* spp. and *Kosakonia* sp. Both Independently and Combined with Fungicides against *Botrytis cinerea* on Strawberries

**DOI:** 10.3390/antibiotics13090912

**Published:** 2024-09-23

**Authors:** Tom E. Schierling, Wolfgang Vogt, Ralf T. Voegele, Abbas El-Hasan

**Affiliations:** 1 Department of Phytopathology, Institute of Phytomedicine, Faculty of Agricultural Sciences, University of Hohenheim, Otto-Sander-Str. 5, D-70599 Stuttgart, Germany; ralf.voegele@uni-hohenheim.de; 2 Agrobiota, Vor dem Kreuzberg 17, D-72070 Tuebingen, Germany; wolfgang.vogt@agrobiota.de

**Keywords:** grey mold, biological control, synergistic effects, fungicide tolerance, pesticide alternatives, Azoxystrobin, copper

## Abstract

**Background:** The ascomycete *Botrytis cinerea* is a major pathogen of strawberry, often causing grey mold and significant yield losses. Its management has largely relied on chemical fungicides, which, while effective, can lead to resistant pathogens and harm to non-target organisms and pose health risks. **Objectives:** This study explored a strategy for minimizing chemical usage by combining biocontrol agents (BCAs) with half-strength fungicide input. **Results:** In vitro results of fungicide-amended culture plates indicated that the presence of 625 µg mL^−1^ Azoxystrobin exhibited no growth inhibition of *T. atroviride* T19 and *T. harzianum* T16 but increased conidial density of T16 by 90%. Copper (750 µg mL^−1^) did not suppress the growth of *T. virens* TVSC or T16 but rather promoted it by 9.5% and 6%, respectively. Additionally, copper increased T16 sporulation by 1.4-fold. Greenhouse trials demonstrated that combining T23 with half-strength Azoxystrobin was as effective as the full dosage in suppressing flower rot. Among the antagonists assessed, *Kosakonia* sp. exhibited the lowest incidence of fruit rot, whereas T23 resulted in a moderate incidence. Moreover, the combination of T16 or *Kosakonia* sp. with half-strength copper was almost as effective as the full dosage in reducing fruit rot. **Conclusions:** Our findings suggest integrating these BCAs in the sustainable management of grey mold in strawberries.

## 1. Introduction

The demand for fresh, high-quality strawberries (*Fragaria ananassa* Duch.) has risen steadily in recent years [1,2,3]. The fruit has long been used for more than just direct consumption. It is also found in yogurts, baked goods, smoothies, and jams. Consumers today are better informed and demand high-quality, nutritionally versatile, and healthy plant-based products [1,2,4,5,6]. This has led researchers and manufacturers to monitor the production, transportation, and marketing of food [2]. Strawberry is a particularly demanding crop and highly susceptible to infection by phytopathogenic fungi such as *Botrytis cinerea* (gray mold), which predominantly proliferates in close proximity to the soil surface [7,8,9]. Consequently, numerous cultivation practices and a high level of plant protection are needed. Although cultivating strawberries in ridges and applying straw before fruit ripening can improve plant health, it does not fully eliminate the risk of pathogen infection. 

*B. cinerea* (teleomorph: *Botryotinia fuckeliana*) is a necrotrophic fungus with a very broad host spectrum of over 200 plant species, producing different disease symptoms, particularly gray mold rot, a devastating disease that makes strawberries inedible [10]. Its mycelium is colored from dirty white to dark grey [11]. In strawberry plantations, the fungus can overwinter in the form of sclerotia and dormant mycelium within plant residues [12]. During the vegetative phase, the fungus produces distinctive gray conidiophores and conidia, which is why it has been designated the colloquial name “gray mold” [13]. Under favorable conditions, the fungus produces one or more large flushes of conidia, which serve as primary inoculum. Conidia are dispersed via air and water spray (rain or overhead irrigation) as well as through contact with infected plant parts [14]. The fungus infects a variety of plant parts, including stems, leaves, flowers, and fruit [15,16,17]. During flowering, germ tubes developing from conidia can directly penetrate into the epidermis of the petals, sepals, stamens, or receptors [18]. Once established in the flower parts, the fungus typically invades the developing fruit, causing fruit rot where secondary spores can be released within a few days [17,19]. Infected strawberries initially exhibit water-soaked spots that subsequently develop into gray mold colonies. The fruit then rots, becomes mushy, and is subsequently coated with a characteristic gray mold [20]. 

Over the past 30 years, the control of *B. cinerea* has been primarily achieved through the use of synthetic chemical fungicides [10]. This approach is not considered sustainable, as it can lead to the development of fungicide-resistant strains. For instance, resistance to benzimidazoles was observed in the *B. cinerea* strains Ben R1 and Ben R2. Furthermore, three *B. cinerea* strains were found to be resistant to anilinopyrimidines [21]. In addition, the discourse surrounding the potential risks associated with plant protection agents and their impact on human and environmental health is becoming increasingly prevalent [22]. Fungicides employed in conventional strawberry cultivation against *B. cinerea* exhibit a number of distinct modes of action. Fludioxonil and Azoxystrobin exert their antifungal effects by inhibiting mitochondrial respiration [23], Cyprodinil acts by inhibiting methionine biosynthesis [24], and Difenoconazole inhibits sterol demethylation [25]. In addition, copper-based fungicides are also approved for use in organic farming of strawberry [26]. These products contain copper hydroxide as the active ingredient and act as a contact fungicide and bactericide [27,28]. The precise mechanism of action remains unclear, but it is hypothesized that copper penetrates the microbial cell, inhibiting enzymatic activities and ultimately leading to microbial death [29]. The chemical compounds employed as fungicides in conventional farming are partly classified as hazardous to human health [30]. When used in accordance with the relevant safety guidelines, the risk of adverse health effects on users and consumers is low [31]. Nevertheless, the possibility of residual contamination cannot be entirely excluded. For instance, the Environmental Working Group (EWG) annually publishes a list of the twelve most pesticide-contaminated fruits and vegetables. In 2023, the EWG identified strawberries as the most contaminated fruit, according to its so-called “Dirty Dozen” list [32,33]. 

An alternative approach to pathogen control involves the use of biological control agents (BCAs) [34]. For several years, numerous fungal and bacterial microorganisms have been the subject of research with regard to their antifungal properties [35,36,37,38,39]. Some BCA-based products such as “Polyversum” (*Pythium oligandrum*), TRICHODEX (*T. harzianum* T39) [40], and “Katayayani *Trichoderma viride*” have already been approved as biological plant protection products. 

Members of the genus *Trichoderma* are among the most extensively studied BCAs due to their different mechanisms of action, which include mycoparasitism [41,42,43,44], the induction of resistance to pathogens in the host, [45,46] and the production of antifungal secondary metabolites [47,48,49,50]. These fungi are also characterized by their ability to degrade cell walls of pathogens [51,52,53]. Furthermore, they are able to compete with other organisms for space and nutrients [54]. A number of the bioactive secondary metabolites produced by *Trichoderma* spp., such as 6-pentyl-α-pyrone, viridiofungin A, harzianic acid, and harzianolide, have been widely studied for use against different plant pathogens [47,55,56,57,58,59]. 

In addition, species of the bacterial genus *Kosakonia* exhibit remarkable genetic diversity and are described as plant growth-promoting agents [60]. In 2018, Lambrese et al. demonstrated that *Kosakonia radicincitans* produces a siderophore that affects mycelial growth and conidia germination of *B. cinerea* [61]. 

A novel approach to reduce the reliance on fungicides involves the integration of BCAs with fungicides in either single or successive treatments [62,63]. This strategy has the potential to markedly reduce the quantity of chemical fungicides utilized [62]. While alternating the treatment of crops with BCAs and fungicides has been investigated for several years, the direct combination of BCAs and chemical fungicides in a single treatment has not been extensively studied [64,65]. The direct combination necessitates a detailed investigation of the compatibility between the BCAs and the chemical fungicides. The integration of *Trichoderma* spp. and *Kosakonia* sp. with chemical fungicides represents a promising sustainable approach to manage *B. cinerea*, provided that the microorganisms are compatible with the chemical fungicides in question. It was shown that *T. asperellum* isolates are sensitive to Azoxystrobin but compatible with copper hydroxide [66,67]. Also, the fungicides Captaf, Thiram, Chlorothalonil, and copper hydroxide were found compatible with *T. harzianum* up to 100 µg active ingredient (a.i.) mL^−1^ [68]. Elad et al. combined *T. harzianum* and the fungicide Ipridione to control *B. cinerea* on cucumber under greenhouse conditions and found the combination treatment to be more effective than either alone [69]. Although Azoxystrobin reduced the conidial germination of *T. asperellum* in vitro, a combined treatment of the latter was found to be more effective against *Sclerotinia sclerotiorum* on lettuce than *T. asperellum* in sole treatment [70]. Barakat and Al-Masri found a combination of *T. harzianum* and the fungicides Cyprodinil and Fludioxonil to reduce disease severity of *B. cinerea* on strawberry, whereas both components in sole treatment showed less efficacy [71]. *Bacillus methylotrophicus* in combination with the fungicide Fluopimomide was shown to be more effective against *B. cinerea* on tomato plants than either treatment alone [72]. 

The combination of BCAs and a chemical agent presents inherent challenges due to the antimicrobial activities of the latter. Therefore, it is essential to evaluate how these components interact with each other and perform in combination [73]. 

The objectives of our study were (1) to investigate the level of tolerance of preselected BCAs (*Trichoderma* spp. and *Kosakonia* sp.) when cultivated on fungicide-amended media, specifically assessing the impact of various fungicides on mycelial growth and proliferation of BCAs in vitro, and (2) to evaluate the efficacy of various combinations of BCAs and reduced levels of chemical fungicides against *B. cinerea* in a greenhouse setting.

## 2. Results

### 2.1. Tolerance of Trichoderma spp. to Various Fungicides in Terms of Growth and Sporulation

**Figure A1:**
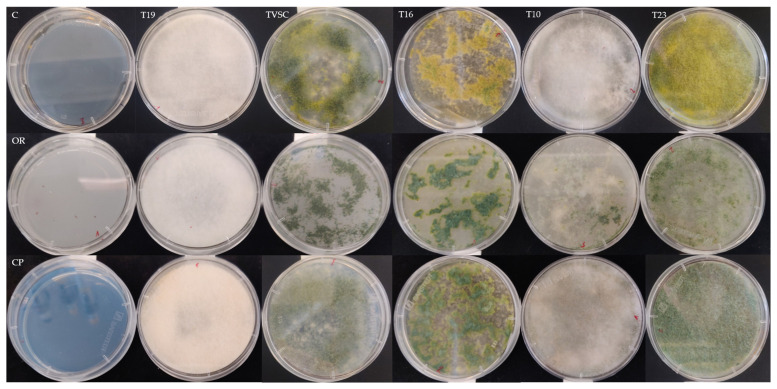
Growth of *Trichoderma* spp. on GM7 culture medium (C) amended with Ortiva (OR) and Cuprozin Progress (CP).

The sterility control (water) showed no mycelial growth on all media. On GM7 without fungicides (control), all *Trichoderma* strains showed no growth inhibition (Appendix A, Figure A1). In contrast, their growth was completely suppressed in the presence of SC (Table 1). Similarly, the mycelial growth of *Trichoderma* strains T10 and T23 was completely inhibited when cultivated on medium supplemented with SW. However, in the presence of the latter fungicide, strain T19 experienced a 70.7% reduction in mycelial growth, while strain T16 showed a moderate growth inhibition (50%). When OR was incorporated into the medium, *Trichoderma* strains reacted adversely compared to the control. While strains T10 exhibited slight (15.8%), T23 moderate (30.3%), and TVSC strong (43.4%) growth inhibition, strains T16 (−1.7%) and T19 (−2.6%) showed no growth inhibition and in fact demonstrated a minimal growth promotion. The presence of CP in the medium induced significant variations in the responses of *Trichoderma* strains. Several *Trichoderma* strains exhibited slight sensitivity to CP, with the mycelial growth of T10, T19, and T23 being inhibited by only 3.6%, 9.7%, and 12.8%, respectively. In contrast, strains T16 (−11.7%) and TVSC (−7.2%) demonstrated growth promotion, showing the highest levels of growth enhancement.

On the other hand, the sporulation of *Trichoderma* strains T10, T16, T19, and T23 was almost completely diminished when exposed to SW. To a lesser extent, strain TVSC showed a strong sporulation inhibition of 71.0% compared to the control. Similarly, sporulation of *Trichoderma* strains showed high sensitivity when subjected to SC. Nevertheless, sporulation of *Trichoderma* strains T19 (59.0%) and TVSC (54.8%) was moderately sensitive to OR.

In the presence of the same fungicide, strains T10 and T23 showed slight sporulation inhibition of 27.3% and 34.5%, respectively. Notably, strain T16 exhibited a significant increase in sporulation (−93.8%) when exposed to OR compared to the control treatment. Under CP treatment, strain TVSC exhibited moderate inhibition of sporulation (51.6%), whereas strain T23 showed negligible inhibition (3.5%). In contrast, strain T10 induced slight promotion of sporulation, with an increase of 9.1%. Remarkably, strains T16 and T19 experienced the highest levels of sporulation enhancement, with increases exceeding 140% compared to the control.

### 2.2. Growth of Kosakonia sp. on Fungicide-Amended Medium

**Figure 1:**
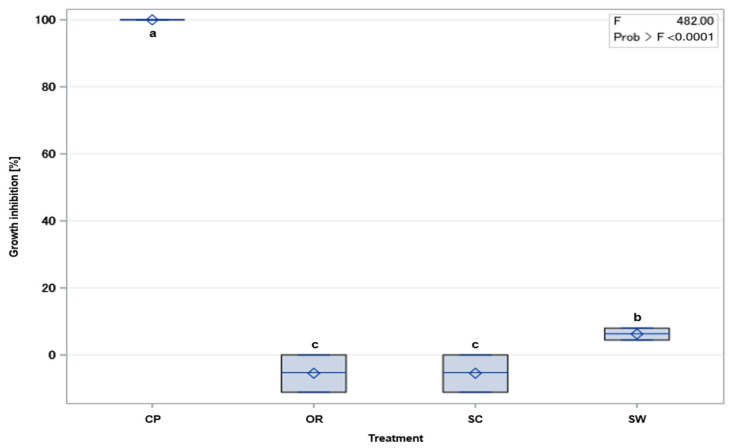
Effect of fungicides Cuprozin Progress (CP), Ortiva (OR), Score (SC,) and Switch (SW) on the growth of *Kosakonia* sp. Diamonds or lines represent mean or median, respectively. Boxes indicate lower and upper quartile. Treatments with the same letter for mean values are not significantly different at a probability level of *p* = 0.05.

On medium supplemented with SW, *Kosakonia* sp. exhibited a growth inhibition of 6.3% (Figure 1). On the other hand, in the presence of SC and OR, *Kosakonia* sp. demonstrated a growth promotion of about 5.5%. Under CP treatment, *Kosakonia* sp. showed a total growth inhibition reflecting a marked decrease in growth compared to the control.

### 2.3. In Planta Assessment of the Potential of BCAs and Fungicide Combinations against Botrytis cinerea

**Figure 2:**
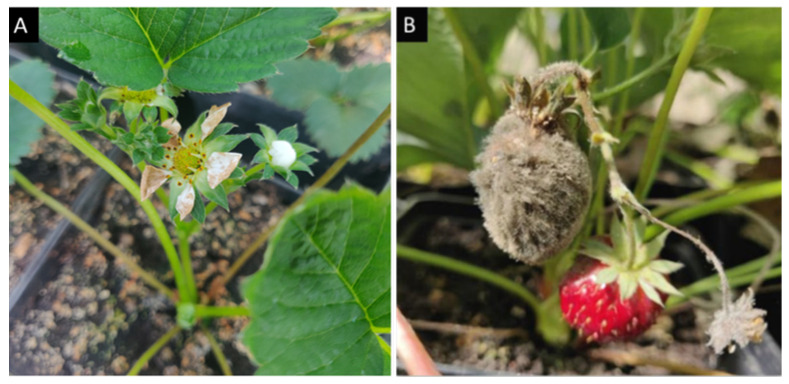
Symptoms on strawberry flowers (**A**) and fruit (**B**) due to *B. cinerea* infection.

The inoculation of *B. cinerea* on strawberry flowers and fruits in the greenhouse was successful. Characteristic symptoms such as gray mold and rot appeared prominently, confirming the efficacy of the inoculation method (Figure 2).

**Figure 3:**
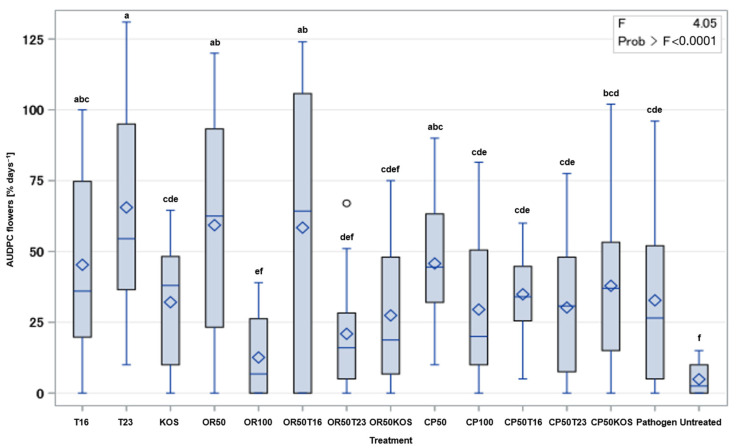
Effect of BCA/fungicide treatments on the infestation of flowers with *B. cinerea*. CP: Cuprozin Progress; OR: Ortiva; KOS: *Kosakonia* sp.; T16: *Trichoderma harzianum* T16; T23: *T. asperellum* T23. Mean values of the AUDPC of 12 replicates per treatment are shown. Diamonds or lines represent mean or median, respectively. Boxes indicate lower and upper quartile. Whiskers are of length 1.5 × inter quartile range. Dots represent outliers. Treatments with the same letter are not significantly different at a probability level of *p* = 0.05.

Among the AUDPC values obtained from three scorings of infested flowers, the control treatment showed the lowest value of 4.9% days^−1^ (Figure 3). Plants treated with antagonists T16 or T23 or the pathogen alone showed comparable and higher levels of disease incidence on the flowers, whereas KOS showed significantly lower disease incidence compared to the other standalone BCA treatments. OR50 caused the highest AUDPC values, indicating the most severe disease incidence. This value, however, differed only insignificantly from OR50T16. In contrast, OR100 showed the lowest AUDPC values, indicating the least severe disease incidence. OR100 exhibited slightly lower AUDPC values compared to OR50T23. However, the latter two treatments shared some overlap in statistical grouping (‘def’ for OR50T23 and ‘ef’ for OR100), indicating that the difference may not be highly significant. On the other hand, OR50KOS had a higher AUDPC value compared to OR100, indicating severe disease incidence. The statistical grouping (‘ef’ for OR100 and ‘cdef’ for OR50KOS) suggests a significant difference in disease incidence between these treatments.

CP100, CP50T16, and CP50T23 treatments exhibited similar AUDPC values, as indicated by their shared grouping (‘cde’), suggesting comparable disease incidence among these treatments. Notably, all these treatments demonstrated significantly lower disease incidence compared to CP50 ‘abc’ and CP50KOS ‘bcd’, indicating that the combination of *Trichoderma* strains T16 or T23 with half the amount of Cuprozin Progress (CP50T16 and CP50T23) is as effective as the full rate of the fungicide (CP100). 

**Figure 4:**
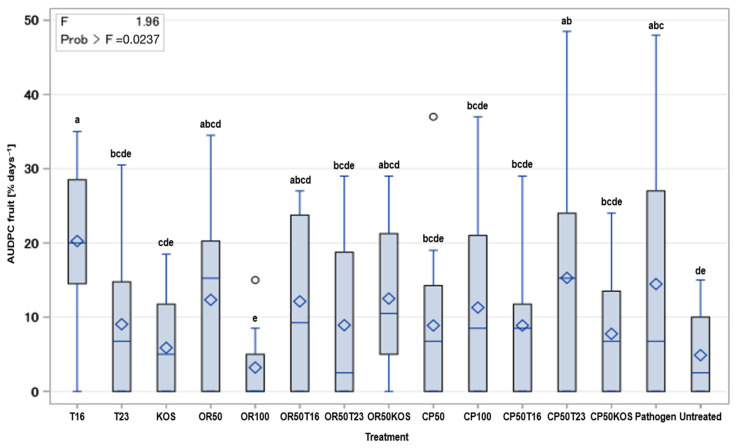
Effect of BCA/fungicide treatments on the infestation of fruit with *B. cinerea*. CP: Cuprozin Progress; OR: Ortiva; KOS: *Kosakonia* sp.; T16: *Trichoderma harzianum* T16; T23: *T. asperellum* T23. Mean values of the AUDPC of 12 replicates per treatment are shown. Diamonds or lines represent mean or median, respectively. Boxes indicate lower and upper quartile. Whiskers are of length 1.5 × inter quartile range. Dots represent outliers. Treatments with the same letter for mean values are not significantly different at a probability level of *p* = 0.05.

When comparing the antagonists without fungicides, T16 showed the highest disease incidence on fruits among all treatments (Figure 4). The pathogen alone also exhibited high disease incidence but slightly lower than T16. 

While T23 treatment resulted in moderate disease incidence, KOS showed the least disease incidence among all antagonist treatments. The statistical groupings confirm significant differences between these treatments. OR100 showed the lowest AUDPC values among treatments of this group and in general, indicating the least disease incidence on fruits. Treatments OR50, OR50T16, and OR50KOS revealed moderate AUDPC values. These three treatments did not significantly differ from each other, as they shared the same significant grouping: ‘abcd’. Moreover, the latter treatments did significantly differ from OR50T23 ‘bcde’. OR100 had lower disease incidence compared to OR50T23. Nevertheless, there is some overlap in groupings ‘e’ and ‘bcde’, suggesting the difference may not be highly significant.

Treatments CP100, CP50, CP50T16, and CP50KOS showed moderate and similar levels of disease on fruits. CP50T23 exhibit a higher disease incidence, showing the most severe disease incidence among the compared treatments. However, significant differences were observed with KOS and OR100 treatments when compared to the pathogen-inoculated control. 

## 3. Discussion

Application of chemical fungicides, particularly copper-based fungicides, in strawberry cultivation raises a significant environmental and consumer concern due to the accumulation of fungicide residues in the soil, flowers, and fruit [74,75,76]. Consequences include a reduction in biodiversity and a potential health risk to consumers [77,78]. Furthermore, frequent applications of chemical fungicides can also result in the development of resistance in pathogens [79]. Environmentally friendly biological alternatives such as BCAs offer effective solutions to minimize or even eliminate problematic of chemical fungicides [80]. Some *Trichoderma* strains are among the best-studied BCAs [81]. The production of antifungal secondary metabolites, the promotion of plant growth, and the induction of resistance mechanisms can collectively reduce the infestation of plants with phytopathogens [46,82,83]. *Kosakonia* sp. has also been shown to reduce infection pressure [84]. Nevertheless, chemical fungicides remain an effective and readily accessible treatment option. However, combining BCAs with existing chemical fungicides may offer alternatives or even superior protection of crops compared to using chemical fungicides alone. This approach can also reduce the reliance on environmentally harmful chemical fungicides [69,70,72,85,86,87]. A key focus of this study was to assess the compatibility of promising BCA strains with conventional chemical fungicides used in strawberry cultivation. Specifically, we evaluated the impact of various fungicides, namely Switch, Score, Ortiva, and Cuprozin Progress, on the growth and sporulation of *Trichoderma* spp. and *Kosakonia* sp. Moreover, combinations of BCAs and fungicides were tested against *Botrytis cinerea* in planta. The results of the present study indicate that the growth of BCAs is either inhibited or promoted to varying extents depending on the fungicide used. Notably, growth of all tested *Trichoderma* strains was significantly inhibited by Switch (937.5 mg L^−1^ Cyprodinyl, 625 mg L^−1^ Fludioxonil), which matched the recommended application rate for controlling *B. cinerea* on strawberry. Barakat and Al-Masri (2017) [71] already demonstrated that *T. harzianum* exhibited significant inhibition rates at concentrations as low as 3.6 ppm Cyprodinyl and Fludioxonyl during growth and 15 ppm during sporulation. Given that the threshold for inhibiting mycelial growth and sporulation of *Trichoderma* spp. is this low, even a combination treatment with 50% of the recommended application rate of Switch with *Trichoderma* spp. seems impractical. The inhibition of growth and sporulation by Score (298 mg L^−1^ Difenocazole) and Switch indicated that all tested *Trichoderma* strains were sensitive to these fungicides. Ortiva (625 mg L^−1^ Azoxystrobin) and Cuprozin Progress (750 mg L^−1^ Copper) treatments on the *Trichoderma* strains T10, T19, T23, and TVSC were found to be variable. Interestingly, the growth and sporulation of strain T16 were observed when exposed to both fungicides. However, Da Silva et al. (2018) [70] found a reduction in spore germination of *T. asperellum* in the presence of 0.36–0.42 µg L^−1^ Ortiva. Ladi et al. (2020) confirmed a tolerance of *T. asperellum* to copper hydroxide [73]. Given this established tolerance, strain T16 presents a promising candidate for combination treatments with both Ortiva and Cuprozin Progress. This synergistic approach could potentially enhance the efficacy of disease control by utilizing strain T16 alongside the protective properties of Ortiva and Cuprozin Progress.

The growth of the Gram-negative bacterium *Kosakonia* sp. was entirely suppressed by Cuprozin Progress (750 mg L^−1^ Copper), attributable to the bactericidal properties of copper [26,27,28,88]. Nevertheless, growth of *Kosakonia* sp. is not entirely excluded at lower concentrations of copper, as used in the CP50KOS treatment. It was demonstrated that BCAs like *Pseudomonas fluorescens* and *Bacillus subtilis* can proliferate in medium containing 138 ppm copper hydroxide [89]. Thus, the CP50KOS treatment was examined in planta. Furthermore, the fungicides Switch, Score, and Ortiva exhibited variable effects on the growth of *Kosakonia* sp. All three treatments did not differ significantly from the control. This lack of significant growth inhibition indicates that *Kosakonia* sp. can tolerate these fungicides and is thus a viable candidate for combination treatments involving Ortiva, Switch, or Score.

This combination of antifungal treatments with different modes of action can also reduce the selection pressure on pathogens, thereby reducing the risk of resistance development [62]. Anand et al. (2010) demonstrated that while an exclusive treatment with *Pseudomonas fluorescens* was insufficient to control powdery mildew (*Leveillula taurica*) and fruit rot (*Colletotrichum capsici*) in chili, a combined application of BCAs with a twofold reduced dosage of the fungicide Azoxystrobin proved to be as effective as the fungicide at the standard dose [62,86]. The results of this study partially support the previously stated findings of Anand et al. (2010) [86], as we were able to demonstrate significant differences in disease incidence between OR100 and OR50KOS treatments on fruit but not on flowers.

Based on the in vitro study, the fungicides Ortiva and Cuprozin Progress were selected for the in planta BCA/fungicide combination trial due to their positive effects on *Trichoderma* strain T16. Strain T23 exhibited a degree of tolerance to both fungicides, with varying degrees of efficacy on mycelial growth and sporulation, respectively. *Kosakonia* sp. demonstrated inhibitory effects in the presence of Cuprozin Progress. However, mixtures with half the application rate of the in vitro test were used in the in planta combination test, which could reduce these effects.

Given that all plants in the greenhouse experiment showed symptoms of gray mold on flowers and fruit, the method of inoculation with *B. cinerea* proved to be successful. On flowers, the untreated control treatment showed the lowest AUDPC value, whereas the pathogen-inoculated control exhibited a significantly higher AUDPC value (Figure 3). Although infection was not completely inhibited in the control treatment, this difference indicates that a clear differentiation can be made between the two treatments. This served as a basis for the evaluation of plant health. The infection observed in the control group with *B. cinerea* can be attributed to the relatively small greenhouse cabin (approximately 20 m^2^) and the airflow generated by the air conditioning system, which facilitated the dissemination of *B. cinerea* conidia through the air [90].

On the other hand, T16 and T23 showed comparable and significantly higher levels of disease incidence compared to the control. Solely the KOS treatment demonstrated significantly lower disease incidence than the standalone treatments. However, the latter treatment did not markedly differ from the pathogen inoculated control. These findings do not rule out the potential of using sole antagonist treatments for controlling *B. cinerea* in strawberries, as an infection of the flower does not necessarily guarantee an infection of the fruit [7,91]. 

Among the Ortiva-containing treatments, OR50 caused the highest AUDPC value, indicating the most severe disease incidence. OR50T16 differed only insignificantly from this value, showing that halving the fungicide concentration had a severe impact on the efficacy of the latter. When used in accordance with the manufacturer’s instructions, Ortiva is a highly effective chemical fungicide. Accordingly, this outcome illustrates the detrimental consequences of non-compliance with the prescribed application rates. A reduction in the application rate can result in a significant intensification of damage to the crop. The cause of this result has not been definitively established in the literature, with the possibility that the pathogen developed resistance more rapidly than previously thought [92]. It is more likely that the OR50 treatment provided a concentration of azoxystrobin too low to prevent germination of *B. cinerea* conidia. It is also possible that the regular formation of new flowers on the strawberry plant may have influenced the results. However, a combination of OR50 and T16 could not compensate for that loss in efficacy. In contrast, OR100 and OR50T23 demonstrated the lowest AUDPC values, with OR100 having a marginally lower AUDPC value, indicating the least severe disease incidence for both treatments. Conversely, OR50KOS showed a significantly higher AUDPC value than the latter treatments, suggesting a more pronounced disease incidence. These results highlight the potential of integrating T23 with markedly reduced dosages of Ortiva (OR50) to significantly achieve robust disease control on strawberry flowers. This strategy offers a suitable alternative to OR100, aligning efficacy with best practices for sustainable pathogen management. These findings are in line with earlier research, which demonstrated that while Azoxystrobin reduced the conidial germination of *T. asperellum* in vitro, a combined treatment was found to be more effective against *Sclerotinia sclerotiorum* on lettuce than the sole *T. asperellum* treatment [69]. 

On the other hand, CP50 and CP50KOS treatments showed higher AUDPC values than CP100, CP50T16, and CP50T23. Interestingly, the latter three treatments belonged to the same statistical grouping, suggesting similar disease incidence among these treatments. This finding indicates that the CP50T16 and CP50T23 treatments are as effective as CP100, supporting the hypothesis that combining CP50 with T16 or T23 maintains the efficacy observed with CP100, thereby offering potential for optimized fungicide use without compromising disease management performance. However, previous studies on the in vitro compatibility of the *T. harzianum* and *T. asperellum* with copper hydroxide have been verified and extended to also be the case in planta [66,67,68]. 

On strawberry fruits, pathogen treatment exhibited high disease incidence. For antagonists without fungicides, all treatments differed significantly from each other, with T23 having moderate and KOS displaying the lowest AUDPC value, overall indicating low disease incidence and being significantly different from the pathogen treatment (Figure 4). These findings indicate that KOS is more effective against *B. cinerea* on fruits than flowers, suggesting *Kosakonia* sp. as a considerable candidate for controlling *B. cinerea* on strawberry fruits. 

OR100 showed the lowest AUDPC values, indicating the least disease incidence on the fruits. On the other hand, OR50T23 had a slightly higher AUDPC value than the latter, with some overlap in statistical grouping. This again suggests that OR50T23 is a promising combination strategy for controlling *B. cinerea* on strawberry fruit. The effectiveness of OR50T23 underscores its potential as an optimal approach to disease management, combining reduced fungicide use with robust disease control.

Regarding the Cuprozin Progress group, all treatments except CP50T23 showed moderate and similar levels of disease on fruit, indicating a general control ability against *B. cinerea*. Furthermore, it was demonstrated that *Kosakonia* sp. was able to multiply and thus develop its effect, likely due to the lower copper hydroxide concentration compared to the in vitro experiment.

It can be assumed that flowers and fruit not directly treated with fungicide may have had a significantly higher probability of being infected with the pathogen. As a potential solution, it may be beneficial to implement more frequent treatments with fungicides or BCA/fungicide combinations. The low infestation levels observed in some treatments could be explained by the insufficient quantity and infrequent application of *B. cinerea*. For instance, Barakat and Al-Masri (2017) treated their test plants in the greenhouse up to three times with *B. cinerea* in order to achieve sufficient infection level [71].

In summary, a combination of T16, T23, or *Kosakonia* sp. with Azoxystrobin or Cuprozin Progress is generally possible and achieved promising results, indicating a significant reduction in grey mold incidence. However, identifying the right concentration of BCA and fungicide in combination and the effect of drench treatment and supplementary spray inoculation before flowering with BCAs is still the subject for further investigations.

Another approach could involve alternating the application of BCAs and fungicides, as the latter does not come in direct contact with BCAs, thereby improving disease control performance. This strategy exploits the different modes of action of both agents, offering a synergistic approach to disease management.

Additionally, the selection of fungicide-resistant *Trichoderma* strains allows for the implementation of a combination treatment comprising BCA and fungicide [62]. This combined approach not only maximizes disease suppression but also mitigates the risk of developing pathogen resistance, leading to more sustainable and resilient agricultural practices.

## 4. Materials and Methods

### 4.1. Micoorganisms and Media

The fungal strains *Trichoderma koningiopsis* T10, *T. harzianum* T16, *T. viride* T19, *T. asperellum* T23, and *T. virens* TVSC and *Botrytis cinerea* were obtained from the Institute of Phytomedicine at the University of Hohenheim (Stuttgart, Germany). *Kosakonia* sp. was provided by Agrobiota (Tuebingen, Germany). All microorganisms were cultivated on glucose-medium-7 (GM7), according to Karlovsky (1994) [93].

### 4.2. Preparation of Conidial Suspensions of Trichoderma spp.

GM7-agar plates were inoculated with an agar plug (5 mm ⍉) of 14-day-old culture of *Trichoderma* spp. The cultures were sealed airtight with Parafilm and incubated for 10 days at 21 ± 2 °C and daylight. After addition of 4 mL of sterile water to the culture, the fungal biomass was then completely removed with a spatula. The resulting suspension was filtered through four layers of sterile gauze (Hartmann, Heidenheim, Germany) to separate conidia from mycelial fragments. The conidial density was determined using a Fuchs-Rosenthal counting chamber (Brand, Wertheim, Germany). 

### 4.3. Evaluation of Growth and Sporulation of Trichoderma on Fungicide-Amended Media

The tolerance of various *Trichoderma* strains (T10, T16, T19, T23, and TVSC) towards the fungicides Cuprozin Progress (CP), Ortiva (OR), Score (SC), and Switch (SW) was evaluated by individual incorporating CP (750 mg L^−1^ Copper), OR (625 mg L^−1^ Azoxystrobin), SC (298 mg L^−1^ Difenoconazole), or SW (937.5 mg L^−1^ Cyprodinil and 625 mg L^−1^ Fludioxonil) into GM7-agar. After autoclaving the GM7, the fungicide solutions were filtered through a 0.2 µM syringe filter (TH Geyer, Renningen, Germany) and added to the medium at approx. 50 °C. All the fungicides are indicated by the manufacturer to be effective (CP, SC, and SW) or partially effective (OR) against *B. cinerea* and were authorized for use on strawberries in Germany at the time of testing. GM7 without fungicide served as control. Each Petri dish (35 mm ⍉) was inoculated with 200 µL of the *Trichoderma* spp. conidial suspensions at a concentration of 5 × 10^4^ conidia mL^−1^. Therefore, the conidial suspensions were pipetted as one droplet in the middle of the prepared Petri dishes. As a control, sterile water was used. All Petri dishes were sealed airtight with Parafilm, randomized, and incubated for 5 days at 21 ± 2 °C and daylight. To record mycelial growth, cultures were photographed, and the mycelial growth was calculated using ImageJ software v. 1.41 (National Institutes of Health, Bethesda, MD, USA). Subsequently, cultures were harvested, the resulting suspension was filtered through four layers of sterile gauze, and the conidial concentration was measured per treatment using a Fuchs-Rosenthal counting chamber. From the mean values of treatments, the inhibition percentage compared to the untreated control was calculated using Abbott’s formula [94]:

$$Inhibition[\%]=\left(\frac{C-T}{C}\right)\times 100$$

where C and T are the radial mycelial growth of *Trichoderma* spp. in the absence and presence of the fungicide, respectively.

### 4.4. Assessing the Effect of Fungicides, Including Cuprozin Progress, on the Growth of Kosakonia

To test the fate of *Kosakonia* sp. on fungicide-containing media, fungicides were added to GM7 at the above-mentioned dosage. The mixtures were then poured into Petri dishes (35 mm ⍉). Then, 200 µL of the *Kosakonia* sp. cell suspension was added to each Petri dish and spread over the entire agar surface. Cultures were sealed airtight with Parafilm and incubated at 30 °C in the dark. Sterile water served as a control. Each treatment comprised three replicates. After 24 h, all cultures were photographed, and the area covered by bacterial colonies was measured using ImageJ v. 1.41.

### 4.5. Effects of BCAs Alone or in Combination with Fungicides on B. cinerea under Greenhouse Conditions

Strawberry plants (cv. Herzle) were planted in pots (2 L) containing a mixture of soil (CLT, Einheitserde Werkverband, Sinntal-Altengronau, Altengronau, Germany) and sterilized compost (1:1; v:v). The plants were fertilized every two weeks with a 0.2% Wuxal^®^ Super (8% N, 8% P_2_O_5_, 6% K_2_O, and micronutrients; Aglukon, Düsseldorf, Germany). In order to evaluate the effectiveness of combining BCAs (T16, T23, and *Kosakonia* sp.) with fungicides, several treatment scenarios were tested (Table 2). These included applying each BCA alone; applying fungicides at full and half dosage; combining each BCA with half of the dosage of a fungicide; and the use of an untreated control. All treatments were inoculated with *B. cinerea*. One group was not inoculated with the pathogen and served as an untreated, healthy control. Each treatment comprised 24 plants placed in individual trays.

Conidial suspensions of T16 and T23 were adjusted to a concentration of 10^7^ conidia mL^−1^. *Kosakonia* sp. inoculum (KOS) was prepared according to the preparation instructions provided by Agrobiota. The supplied dry granules were diluted with sterile water to achieve a final concentration of 10^9^ cfu mL^−1^. To prepare the full dosage CP treatment (CP100), 3 µL ml^−1^ commercial CP was added to 240 mL sterile water. The preparation of the half-dosage CP treatment (CP50) was achieved by using half the amount of the fungicide. The combination treatments CP50T16, CP50T23, and CP50KOS were prepared by incorporating T16, T23, or KOS into 240 mL filtered, sterile CP50, respectively. A similar protocol was used to prepare the treatment combination with OR. For each treatment, 0.02% BREAK-THRU S 301 (Evonik Industries, Essen, Germany) was added after filtration through a 0.2 µm microfilter.

*B. cinerea* inoculum was prepared from GM7 plates that exhibited heavy sporulation. The concentration of conidial suspension was adjusted to 10^5^ spores mL^−1^. Strawberry plants were sprayed with the relevant antagonist until runoff (~5 mL per plant) using a conventional sprayer. 

Trays were randomly distributed on tables in the greenhouse at 30 ± 5 °C. Relative humidity was set to 85% for the first 24 h using an air humidifier (Condair, Norderstedt, Germany). After 7 days, plants were sprayed again with the respective antagonists. Two days later, plants were inoculated with *B. cinerea* (~5 mL per plant). To avoid cross-contamination, control plants were maintained separately from inoculated ones. Therefore, control plants were removed from the greenhouse cabin for the time of pathogen inoculation. The trays were randomized in the greenhouse. Relative humidity was again set to at least 85% for 72 h. Disease assessment was carried out 14, 21, and 31 days after inoculation with *B. cinerea*. The total number of flowers, the number of flowers with visible symptoms, the total number of fruits, and the number of fruits with visible symptoms were recorded for six identical plants in each treatment. The area under disease progress curve (AUDPC) was calculated using the formula:

$$AUDPC=\overset{n-1}{\underset{i=1}{\sum }}\left(\frac{y_{i}+y_{i+1}}{2}\right){\left(t_{i+1}-t\right)}_{1}$$

where *t* is the time of each assessment, *y* is the percentage of infested plant parts in each assessment, and *n* is the number of readings. The variable *t* stands for days [95]. In addition, the results of the AUDPC calculation were presented as inhibition compared to the control inoculated with the pathogen.

### 4.6. Statistical Analysis

Statistical analyses were performed using the GLM procedure in the SAS software (version 9.4). A one-factorial analysis of variance (ANOVA) or two-factorial analysis of variance (ANCOVA) and a least-significant difference test (LSD) were used to compare treatment means. Differences between treatments were set to a probability of *p* = 0.05.

## 5. Conclusions

The use of chemical fungicides in strawberry cultivation raises environmental and consumer health concerns due to residue accumulation, biodiversity reduction, and the development of pathogen resistance. This study explored the potential of integrating preselected BCAs like *Trichoderma* strains and *Kosakonia* sp. with chemical fungicides to mitigate these issues. Our findings demonstrate that combining BCAs with lower doses of fungicides such as Azoxystrobin and Cuprozin Progress can effectively reduce disease incidence without compromising efficacy. Treatments like OR50T23 and CP50T16 were shown to be as effective as their full-strength counterparts, OR100 and CP100, respectively. *Kosakonia* sp. was particularly effective in controlling *B. cinerea* on strawberry fruits. In conclusion, integrating BCAs with reduced fungicide concentrations presents a sustainable and effective strategy for disease management in strawberry cultivation. This approach reduces chemical usage and addresses both environmental and economic concerns. Further research should aim to optimize these combinations for broader agricultural applications.

## Figures and Tables

**Table 1:** Effect of different fungicides amended in the culture medium (GM7) on the mycelial growth and sporulation of *Trichoderma* spp. Treatments with the same letter for mean values are not significantly different from each other at a probability of *p* = 0.05.

Treatment		Inhibition [%]		
		Mycelial Growth		Sporulation
Control	*Trichoderma* spp.	0	^def^	0
SW	T10	100	^a^	99.6
	T16	50	^abcd^	84.4
	T19	70.8	^ab^	93.2
	T23	100	^a^	96.6
	TVSC	54	^abc^	71
	water	100	^a^	100
SC	T10	100	^a^	80.8
	T16	100	^a^	98
	T19	99.7	^a^	90.7
	T23	100	^a^	99.9
	TVSC	100	^a^	100
	water	100	^a^	100
OR	T10	15.8	^cdef^	27.3
	T16	−1.7	^def^	−93.8
	T19	−2.6	^def^	59
	T23	30.3	^bcdef^	34.5
	TVSC	43.4	^bcde^	54.8
	water	100	^a^	100
CP	T10	3.6	^cdef^	−9.1
	T16	−11.7	^f^	−140.6
	T19	9.7	^cdef^	−148.5
	T23	12.8	^cdef^	3.5
	TVSC	−7.2	^ef^	51.6
	water	100	^a^	100

**Table 2:** Graphical display of the combined and standalone treatments with BCAs and fungicides. CP: Cuprozin Progress; OR: Ortiva; KOS: *Kosakonia* sp.; T16: *Trichoderma harzianum* T16; T23: *T. asperellum* T23.

Treatment	Pathogen	Fungicide Applied Rate [%]		BCAs		
		CP	OR	KOS	T16	T23
Untreated cont.						
Pathogen cont.	x					
T16	x				x	
T23	x					x
KOS	x			x		
CP100	x	100				
CP50	x	50				
CP50T16	x	50			x	
CP50T23	x	50				x
CP50KOS	x	50		x		
OR100	x		100			
OR50	x		50			
OR50T16	x		50		x	
OR50T23	x		50			x
OR50KOS	x		50	x		

## Data Availability

Data are contained within the article; further inquiries can be directed to the corresponding authors.

## Appendix A

*Trichoderma* spp. grown on GM7 and fungicide (OR and CP)-amended GM7 for 5 days at 21 ± 2 °C and in daylight.
